# Interactive effects of mating receptivity and courtship pheromones on the scent preferences of female red-legged salamanders

**DOI:** 10.1371/journal.pone.0343685

**Published:** 2026-03-31

**Authors:** Christy L. Kunkel, Karen M. Kiemnec-Tyburczy, Damien B. Wilburn

**Affiliations:** 1 Department of Biology, John Carroll University, Cleveland Heights, Ohio, United States of America; 2 Department of Biological Sciences, California State Polytechnic University, Humboldt, Arcata, California, United States of America; 3 Department of Chemistry and Biochemistry, The Ohio State University, Columbus, Ohio, United States of America; Sathyabama Institute of Science and Technology, INDIA

## Abstract

The behavioral and endocrine responses elicited by pheromones are highly dependent on the sex and physiology of the receiving individual. In the red-legged salamander (*Plethodon shermani*), male courtship pheromones influence female mating behavior and regulate the timing of courtship. Pheromones also modulate female feeding behavior and scent preference in this species, but little is known about how the physiological state of females may influence their olfactory preferences. The aim of this study was to use laboratory trials to investigate whether differences in female receptivity influence the effect pheromones have on female scent preference. Our first experiment showed that pheromone treatment significantly increased the time females spent on male-scented substrate compared to both female scent and water. In a second experiment, female *P. shermani* with differing levels of mating receptivity were treated with either saline or pheromone and assayed for the relative time spent on each of three scents (male scent, food scent, and water). When females were treated with saline, their preference for male scent was positively correlated with mating receptivity. Application of pheromone also increased preference for male scent, but this effect was greater in females with lower receptivity. Pheromone treatment also decreased preference for food scent and was significantly pronounced in females with higher receptivity. These results suggest that courtship pheromones in *P. shermani* may have dual roles in regulating female preferences based on baseline mating receptivity, further suggesting a complex interplay between male courtship pheromones and female preference dynamics.

## Introduction

Sexual reproduction is a principal source of genetic recombination for most animals, facilitating rapid adaptation to complex environments and purging of deleterious alleles [1,2]. At all stages of reproduction, from courtship initiation through fertilization, male- and female-specific chemicals can be transferred that modify behavior, physiology, and fitness in the other sex. In the last five decades, multiple vertebrate chemical signals have been purified and demonstrated to elicit behavioral and endocrine effects, justifying their classification as pheromones [3–5]. While most characterized invertebrate pheromones are volatile organic compounds, the majority of characterized vertebrate pheromones are water soluble peptides or proteins [6].

Recent work on the effects of pheromones has just begun to investigate how the physiological state of the receiver influences their response to pheromones in vertebrate systems. In mice, females of different reproductive states (estrus or diestrus) respond differently to male pheromones [7]. The sensitivity of fish to sex pheromones can be enhanced by hormonal changes, which may in turn be modulated by social interactions (reviewed in Bowers et al. [8]). Hormones change sensory systems during reproductive cycles in African cichlid fish and can increase sensitivity of the olfactory system to courtship signals [9]. However, little is known about how differences in physiological state of the receiver impact behavioral responses to chemical cues in amphibian taxa.

Pheromone communication is common to all three amphibian clades (frogs/toads, salamanders/newts, and caecilians), underpinned by deep ancestry, broad conservation, and extensive duplication of pheromone genes such as sodefrin precursor-like factor (SPF) [10,11]. Little is known about pheromone signaling in caecilians [12]. While vocal communication is extensive in frogs and toads [13], only recently have some anuran pheromones been biochemically and functionally characterized [14,15]. Mostly non-vocal salamanders and newts, by contrast, are classic models of pheromone signaling and rely extensively on chemical cues for foraging, predator avoidance, and social interactions that include reproduction, mating, and courtship [5]. Male plethodontid salamanders use non-volatile protein pheromones to alter female mating behavior and improve reproductive success [16]. Prior to the courtship season, plasma androgens rise and induce hypertrophy of a submandibular mental gland [17] that, once fully developed, synthesizes proteins that function as courtship pheromones [18]. Multiple gene families have been co-opted as pheromones from sperm homologs [11], with most (if not all) of these genes experiencing bursts of rapid evolution through gene duplication and positive Darwinian selection [18–20].

The composition and role of these pheromones have been most extensively examined in the red-legged salamander, *Plethodon shermani* [21–23]. These salamanders produce a cocktail of proteins that have been shown to reduce courtship duration. Detailed biochemical characterization through transcriptomic, mass-spectrometry-based proteomics, and NMR-based structural analysis support that the two most abundant proteins in the *P. shermani* mental gland are related to IL-6 cytokines and three-fingered proteins [22,24]. Male plethodontid salamanders do not restrain females during courtship, and females may flee and/or abandon the courtship at any time [25]. Therefore, the male must maintain the focus of the female during courtship, and we postulate that the mental gland pheromones facilitate this process. Female *Plethodon* are generally the more selective sex and likely benefit from stringently screening potential mates [25]. Population densities are often greater than 2.5 salamanders per square meter [26], and females may constantly assess potential mates to replace previously acquired sperm. Although the natural number of matings is not known in *P. shermani,* natural clutches of other plethodontid species are usually sired by at least two males [27] but pairs mate as many as 15 per season in laboratory trials [28].

In *P. shermani*, populations consist of females of variable receptivity to mating during each long breeding season (mid-July to early-October). Females have biennial reproductive cycles, and only about half of females are in reproductive condition during any given courtship season with highly varying levels of gravidity [29]. Given that a population of female *P. shermani* are of variable receptivity, female interests are likely to vary between time spent foraging and seeking mating opportunities across breeding season [30,31]. As feeding and reproduction are often considered conflicting investments on female time and energy [32], pheromone signals that shift female behaviors towards mating may be favored if they increase a male’s reproductive success. Studies have shown that male courtship pheromones lower female *P. shermani* feeding activity [31] and increase their preference for a male scent over a non-sexual olfactory stimulus [30]. But it is not known whether courtship pheromone effects can vary depending on a female’s receptivity state in this species. In this study, we investigated how male courtship pheromones and female receptivity could jointly affect female scent preference. We designed a simultaneous choice set up that we used to test the hypothesis that females in reproduction condition display a behavioral preference for scents from males or females (Experiment 1). Once we had verified the behavioral assay experimental design was effective, we used the same experimental setup to investigate the time females of varying mating receptivity levels each spent on multiple types of scented substrates with and without prior application of male courtship pheromones (Experiment 2). Female scent preferences for three different substrates (a water control, a food scent, and male scent) were measured to explore the effects of pheromone on the female’s conflicting interests. We hypothesized that (1) females that have lower receptivity to mating are less likely to spend time investigating a male’s scent and more time on food scent and (2) the male courtship pheromones may have more pronounced effects on the scent preferences females with lower receptivity because these females may be less motivated to seek out males.

## Materials and methods

### Animal capture and maintenance

Methods and animal care described herein were approved by Oregon State University’s Institutional Animal Care and Use Committee (ACUP 3007 and 4053 to L.D. Houck). Using appropriate collecting permits, adult *P. shermani* in reproductive condition were collected in August 2012 and 2013 from a single locality in Macon Co., North Carolina, USA (35° 10’ 48” N, 83° 33’ 38” W) and transported to the Highlands Biological Station (Highlands, NC) where they were maintained at 70% humidity and temperatures between 17–20°C, housed in a plastic box (17 cm x 9 cm x 13 cm) lined with a damp paper towel, and provided with an additional crumpled paper towel for refuge. Screening trials were conducted at the Biological Station. Animals used in experiments were shipped to Oregon State University, Corvallis, OR USA in August 2012 or 2013 and kept at similar conditions, on a late August North Carolina photoperiod. Each animal was housed individually in a clear plastic box as described above; boxes were cleaned weekly. All salamanders were fed two waxworms (*Galleria mellonella*) each week. Experiments took place in October, within the breeding season of *P. shermani*. Both experiments took place in the same year and season as when the animals were collected (2012 or 2013). The authors were blind to treatment groups during animal observations.

### Female receptivity screening trials

Approximately 60 adult male and 50 female P. *shermani* were randomly paired in a series of screening trials to determine their mating propensity in a laboratory setting. All animals used in the screening trials and subsequent experiments were in reproductive condition: males had well-developed glands and females were visibly gravid. Pairs were set up for two screening trials per animal. Each female was paired with a new, unique male at each trial. Each pair was placed in a clean box lined with a single moist paper towel and left overnight. The following morning, each animal was returned to its original box and each screening box was scored for the presence or absence of a spermatophore base. The presence of a spermatophore base indicated a successful courtship that resulted in sperm transfer (female had picked up the sperm cap into her cloaca, leaving the gelatinous base behind on the substrate). A “receptivity” score (R-score) was calculated as the ratio of times a female mated to the number of screening trials in which she participated (0 ≤ R ≤ 1). If a female successfully mated in two successive trials, she was assigned “high receptivity” with R = 1. Females that mated once in the two trials were assigned R = 0.5. If a female did not mate in the first two trials, she was presented a max of two additional to mating opportunities for possible R-scores of 0.0, 0.25, or 0.33. All scores less than 1 (i.e., 0.25, 0.33, and 0.5) were designated “low receptivity.” These screening trials occurred at least two weeks before the experiments described below (i.e., all screening trials were completed by mid-September). Experiments 1 and 2 were conducted at least two weeks after screening to allow females time to ‘regain’ receptivity that may have been transiently reduced after mating (as shown by [33]). Based on observations that these animals have an extended breeding season [29] and that other species in the Plethodontidae can mate more than a dozen times in laboratory trials [28], we assumed that these screening matings would not decrease a female’s future interest in mating (and therefore her investigation of male scent) within the same breeding season.

### Scent wash preparation

For use in Experiment 1, body wash was collected from seven individuals of each sex. Each animal was placed in a small plastic container with 45 ml of dechlorinated water and left overnight. The water level was sufficient to cover the feet and ventral surface but not submerge the animal’s body. Wash from each container was pooled to create two homogenous sex-specific mixtures, diluted with 100 ml dechlorinated water, and divided into 75 ml aliquots for use in subsequent trials. In Experiment 2, test scents included body wash from reproductive males (from the same preparations as Experiment 1) and waxworms. To create the waxworm (*Galleria mellonella*) food wash we used a method similar to that of Vaccaro et al. [30], 120 waxworms were submerged in water and allowed to soak overnight (100 ml per 60 waxworms). The waxworm body washes were pooled, diluted with 100 ml of dechlorinated water, and divided into 75 ml aliquots. All scent solutions were stored at −20°C for up to 2 weeks and then thawed for 2 hours before experimental trials, preventing any degradation that might occur during multiple freeze/thaw cycles.

### Pheromone preparation

*Plethodon shermani* whole pheromone extract was prepared using methods described by Rollmann et al. [22]. Briefly, adult male *P. shermani* were anesthetized in a mixture of 7% diethyl ether in water, and the mental gland was surgically removed from the dermis. While diethyl ether is not generally recommended for anesthesia of terrestrial salamanders, its use in this case was justified because the other evaluated anesthetics – MS-222 (tricaine methanesulfonate) (e.g., Novarro et al. [34]) and benzocaine (e.g., Cecala et al. [35]) – are acetylcholine agonists. Because pheromone proteins are released from vesicles via acetylcholine signaling, exposure to these agents interferes with the mental gland pheromone extraction and thus cannot be used.

Following surgery, each male was placed in a clean box and the chin rested on a small piece of gauze containing an antibiotic ointment. The mental gland is found immediately under the 2–3 cell layers of the epidermis, and its removal is minimally invasive. Pheromones were extracted from the excised mental glands by incubation in 0.8 mM acetylcholine chloride in Amphibian Ringer’s Solution for ~60 minutes. The solution was centrifuged at 10,000 *x g* for 10 minutes to remove the cellular debris, the supernatant collected, and the centrifugation repeated before storage of supernatant at −80°C. The pheromone extract was concentrated using a YM-3 Centriprep (Millipore, Billerica, MA) and standardized to 2 mg/ml in 0.5X phosphate buffered saline (0.5X PBS).

### Experiment 1: Pheromone effects on sexual scent preference

**Fig 1:**
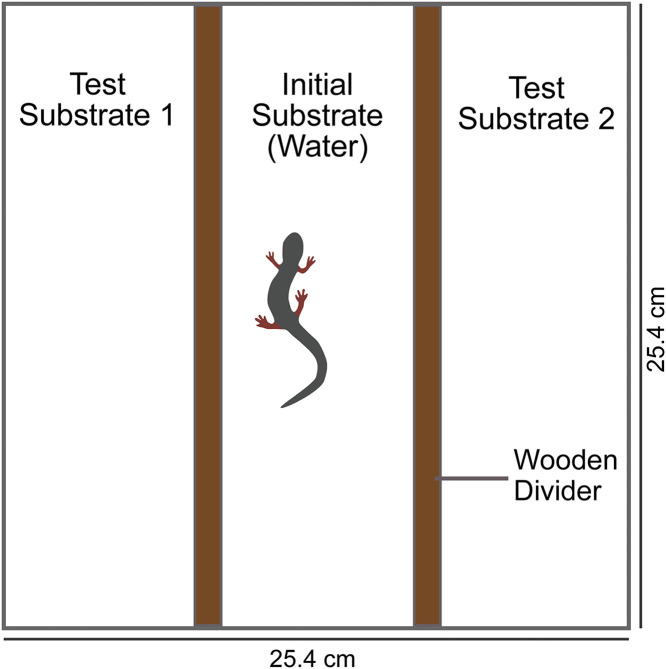
Schematic of the experimental chambers used for female scent preference trials with dimensions in millimeters (mm). The snout to vent length of a typical adult *P. shermani* is ~ 50-60 mm. In the first experiment, the test substrates included male and female *P. shermani* body washes; in the second experiment, the test scents were male *P. shermani* and food (waxworm) body washes.

Gravid females (*n* = 20) that had mated at least once in screening trials were randomly assigned (with respect to screening receptivity score) to participate in this simultaneous choice experiment. The bottoms of square, plastic bioassay boxes (241 x 241 x 20 mm) were lined with three equal-sized strips of paper towel (65 mm x 241 mm) each separated by a 15 mm gap. The paper towel in the center of each box was dampened with dechlorinated water. Each of the paper towels on the sides of the box were moistened with ~7.5 ml of one of the scent washes (male or female scent) to making substrate saturated with a test scent (Fig 1). The placement of each scent (right or left side) was randomized across trials. Wooden barriers were used to confine a female to the center of the box before each trial began. These barriers were created by placing two wooden dowels (1.6 cm x 1.6 cm x 22.8 cm) in each box, one on either side of the central towel. To ensure that scent was not transferred on wood, barriers were used for the same treatment towel in each trial (e.g., either separating water and female scent, or water and male scent in this experiment).

In each box, a female was confined in the center of the box (between the wooden barriers) and allowed to acclimate to the new environment for 10 min. After acclimation, 4 μL of treatment was pipetted to the nares of the female. The treatment a female received was either (a) whole pheromone extract, or (b) a saline vehicle control (0.5X PBS). An additional 10 min were allowed for the treatment to take effect before the barriers were removed and the female was free to explore the box. Trials lasted one hour; five animals in five boxes were observed by a single observer during each trial (4 observers total). Visual scans were made every min for each box. The observer recorded the location of a female’s position in the box; left, middle, or right, but was blind to the locations of the scent treatments. If a female was straddling two towels, her position was recorded as the towel towards which her head was orientated. Observations were conducted between 2100 h and 2300 h EDT at Oregon State University on October 2, 2012.

### Experiment 2: Pheromone effects on scent preference of females of variable mating receptivity

The females (*n* = 40) used in this experiment were a randomly selected subset of those that had mated at least once during screening trials, with receptivity varying from R = 0.25 to 1. Each female was fed a single waxworm three days before each of their behavioral trials to standardize hunger levels. As in Experiment 1, each female was placed in a box lined with three strips of moistened paper towel. In this experiment, however, the two test scents were body wash and waxworm (food) wash. Wooden barriers were placed between towels to confine the female to the center towel, moistened with water, before the experiment began. Females were allowed 10 min to acclimate, and then 4 µL of treatment (saline or whole pheromone extract) was applied to the nares of each female. Females were allowed another 10 min to settle after pheromone treatment before barriers were removed and their position recorded by observers for one hour in the same way as Experiment 1.

Females were separated into two groups and the order in which they received each treatment was randomized. One week later, each female was given the other treatment solution (pheromone or saline) and her scent preferences were recorded in this trial. All the hour-long observations took place between 1800 h and 2200 h PDT at Oregon State University (Corvallis, OR) on October 2–9, 2013.

### Statistical analysis

All data were analyzed by logistic regression with multinomial distributions using the multinom function in the R package nnet [36]. The response variable was the frequency each female was observed on each substrate (observed every min for one hour), and independent variables included female receptivity (proportion of the number of successful matings to number of mating opportunities), pheromone application (saline or whole extract), and the interaction term between receptivity and pheromone. Main effects and interaction terms were sequentially added to models and statistical significance evaluated by likelihood ratio tests. Post-hoc tests for comparison of effects between levels and across variables were performed using Z-tests between log-odds ratios in R. Standard errors calculated from the cumulative model containing both main effects and the interaction term, with Bonferroni correction to adjust for multiple comparisons (for eight comparisons, *p* = 0.00625). All raw data are available on Dryad (https://doi.org/10.5061/dryad.cvdncjtfz).

## Results

### Experiment 1: Pheromone effects on sexual scent preference

**Fig 2:**
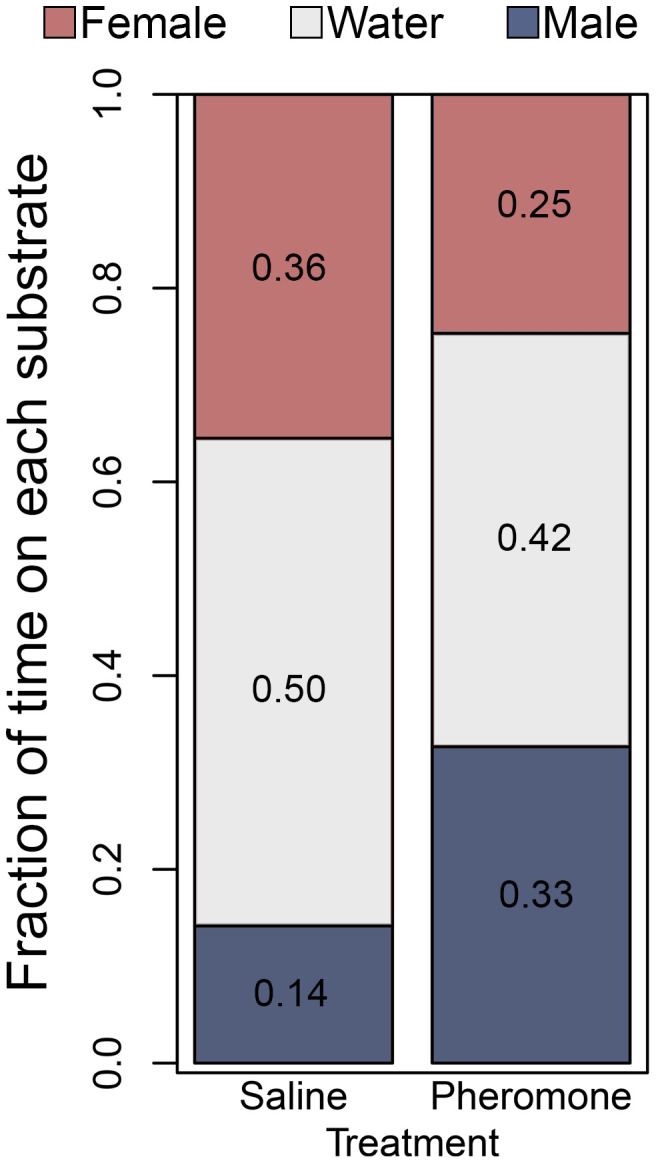
Effect of pheromone treatment on female scent choice for body scent washes from Experiment 1. Bar plots represent mean fraction of the time all females spent on each of the three substrate choices (water, male scent, or female scent) in simultaneous choice experiment.

In Experiment 1, female salamanders were treated with whole pheromone extract or a saline control, and their scent preference for adult male and adult female body washes was assessed. When pheromone was applied to female salamanders, they spent significantly more time on male scent (~2.7X relative to water; *t*-value = 6.45, *p* < 10^−5^), but did not significantly alter the time they spent on female scent (*t*-value = −1.45523, *p* = 0.081). These da*t*a support that *P. shermani* can distinguish between male and female body washes, and that courtship pheromones increase female preference for male scent (Fig 2).

### Experiment 2: Pheromone effects on scent preference of females of variable mating receptivity

**Fig 3:**
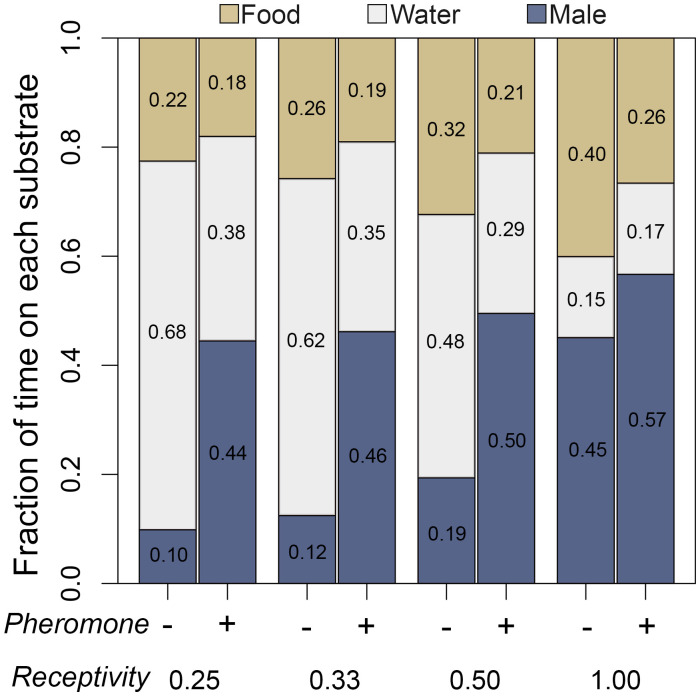
Effect of pheromone treatment and mating receptivity on female scent choice from Experiment 2. Bar plots representing the mean fraction of time females with four different receptivity scores (0.25, 0.33, 0.5, and 1.0) spent on each of the three substrate choices (water, male scent, food scent) when treated with either saline (-) or whole pheromone extract (+).

In Experiment 2, females of varying receptivity were treated with a pheromone extract or a saline control before presented the choice of a male salamander scent (adult male body wash) and a food scent (waxworm body wash). Female receptivity level (determined by screening prior to scent choice trials), pheromone treatment, and their interaction term were all significant (*p* < 10^−7^) variables in the log-linear models. Both application of pheromone and increased female receptivity were positively correlated the proportion of time spent on both food and male scents relative to water (Fig 3). Application of pheromones to low receptivity females increased the proportion of time they spent on the food scent relative to water but did not significantly change the food scent preferences of highly receptive females (Table 1). When the interaction term was included, the model supported that application of pheromone increased the proportion of time low receptivity females spent on male scent relative to water (Table 1). Pheromone application had no significant effect on the scent preferences of highly receptive females. Together, these data indicate that application of pheromone increases interest in male scent by females with low receptivity; however, as receptivity increases, the relative preference for male scent is not altered by pheromone treatment. Highly receptive females spend significantly more time investigating scents of both types.

**Table 1:** Estimated differences in female scent preference for male scent compared to water when females of varying receptivity levels were treated with pheromones (inferred from the logistic regression model).

Comparison groups		Male scent		Food scent	
		Log-odds	*p*	Log-odds	*p*
Saline Low receptivity	Pheromone Low receptivity	0.663	**0.011**	2.758	**<10** ^**−10**^
Saline High receptivity	Pheromone High receptivity	−0.528	0.089	0.110	0.39
Saline Low receptivity	Saline High receptivity	2.785	**<10** ^**−10**^	4.048	**<10** ^**−10**^
Pheromone Low receptivity	Pheromone High receptivity	1.595	**9.0x10** ^**-5**^	1.400	**3.1x10** ^**-4**^

## Discussion

Our study demonstrates an interplay between male courtship pheromones and female receptivity in the regulation of female scent preference in salamanders. Specifically, we first showed that females can discriminate between scents of different sexes and subsequently that application of male courtship pheromones increases female preference for male scent over food scent. These results are similar to those found by Vaccaro et al. [30]. In that study, male courtship pheromones affected female feeding activity and increased their preference for male scent over a non-sexual stimulus. Feeding was suppressed and females spent more time in the vicinity of male scent over water but showed no difference between food and water. In the current study, females were allowed to choose to occupy regions of the experimental chamber with three different substrates simultaneously, providing a stronger comparison and evaluation of how pheromone application affects female preferences. In addition, we quantified the level of mating receptivity for females in the second experiment, allowing us to investigate whether pheromones elicit different effects from receivers with different physiological states. We found differences in pheromone effects and showed that application of pheromone to low receptivity females increased the preference for both male and food scents, but that this effect was not observed in females with high receptivity.

Our results indicate that male *P. shermani* courtship pheromones – which have been well-documented to affect courtship duration (reviewed in Siegel et al. [37]) – also have other behavioral effects. On the proximate level, these pheromones are multi-protein blends, but their numerous effects may be mediated by a common neurological circuit. Male *P. shermani* deliver pheromones by tapping the mental gland to the female’s nares, allowing the pheromones to be detected by the accessory olfactory system and indirectly regulate female physiology [38]. But, in many other plethodontid lineages, males deliver pheromones by scratching the dorsum of the female and rubbing their glands on the abraded region of skin [16]. The bioactive component of the pheromone blend that is eliciting the change in scent preference has yet to be characterized. The full composition of the mental gland pheromones is only known for a few plethodontid species, but biochemical characterization has revealed the presence of peptide hormone-like proteins in the pheromone blend. In *Desmognathus ocoee*, the pheromone extract includes proteins derived from gene paralogs of glucagon, insulin, and leptin – three hormones critical to maintaining blood glucose levels and controlling hunger response in vertebrates [19,39]. While the exact functions of these hormone-like peptides remain unknown, given the known function of their gene families (metabolic control), it is probable that these putative pheromones function as hormone agonists/antagonists to control female feeding behavior or motivation to feed. This may create a false sense of satiation in the female and decrease interest in food. Despite the evolution of different pheromone delivery modes across plethodontid salamanders, it is possible that the downstream effects on female physiology induced by male pheromones have been conserved over evolutionary time. This would be consistent with our current findings in *P. shermani*, where pheromones are delivered via olfaction and stimulate the central nervous system [38].

In *P. shermani*, courtship pheromones in the mental gland are generally delivered to the female after the male has already performed several courtship behaviors, suggesting that changes in the pheromone effects on the female scent preferences may serve as a secondary tactic to increase insemination success after a female has engaged in preliminary courtship. During courtship, however, pheromone application may cause less receptive females to remain in courtship until mating occurs or keep more receptive females focused on the courting male rather than leaving to engage in other behaviors such as foraging. The proposed mechanism of swaying female attention towards courting rather than feeding may represent an example of sexual conflict: females bear an associated cost (reduced energy acquisition) from increased response to male pheromones [40] while males gain increased reproductive success. It is often difficult to provide unequivocal support for cases of sexual conflict, due to the challenge of addressing how natural selection balances the increased costs to females associated with sexual selection [24,41–43]. In the case of *P. shermani*, further research is needed to determine whether reductions in foraging time may impact a female’s fitness. Because we demonstrated that application of pheromone to low receptivity females increased the preference for both male and food scents, we hypothesize that after the male leaves, residual effects from the pheromone may increase subsequent feeding behavior. This increased feeding may lead to better maturation of ova and an increased number of progeny for the male in the following courtship season. This is but one of many possible explanations for the interesting dichotomous pheromone functions identified.

Recent work in both invertebrates and vertebrates reveals that the same pheromone can elicit different responses based on the age [44], dietary status [45], and mating history of the receiver [7,46]. Most of these types of studies, however, have focused on invertebrate taxa, especially insects (e.g., Xu et al. [47]). Our results add to this growing body of research by demonstrating that previous mating receptivity influences a female vertebrate’s response to courtship pheromones. Our results suggest that researchers should carefully consider what time of year and breeding season they are conducting their studies because those involving conspecific scent preference may be impacted by the physiological state and therefore motivation to investigate scent preferences [9]. For practical reasons, experiments in this report were performed near the end of the breeding season which may also have impacts on the results. As with insects, further investigation of the effects of courtship pheromones on females of varying gravidity and receptivity by correlating changes in behavior with neurophysiological responses will help us better understand the dynamic nature of chemical communication.
